# Supplementary material for: Grape SnRK2.7 Positively Regulates Drought Tolerance in Transgenic Arabidopsis
Source: Int J Mol Sci. 2024 Apr 18;25(8):4473. doi: 10.3390/ijms25084473 (PMC11049990; doi:10.3390/ijms25084473)
Supplement: Supplementary file 1 [file ijms-25-04473-s001.zip › ijms-2938882-supplementary.pdf]

## Supplementary Figure legends

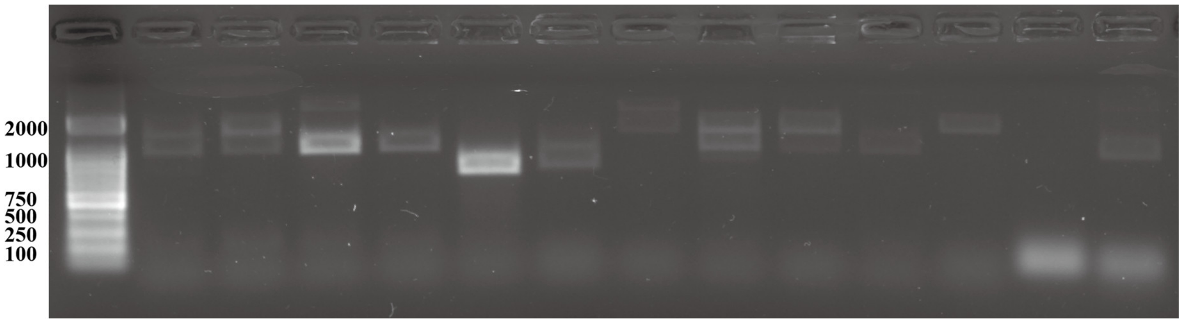


**Figure S1.** PCR strip of escherichia coli liquid of some AD library vectors.The first column is the Maker band, and the rest of the columns are the bacteriophage P bands for some of the different genes obtained by hybridization from the AD library.

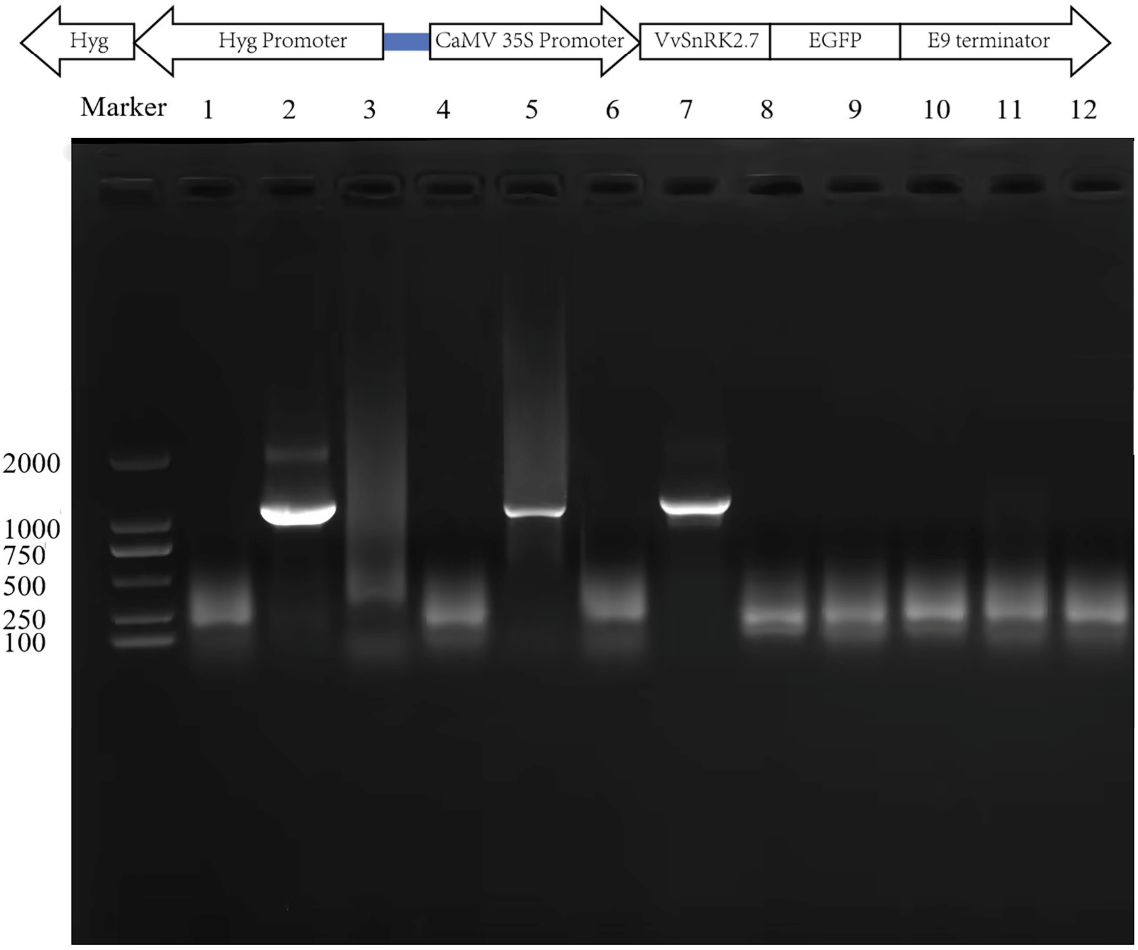


**Figure S2.** Identification of transgenic *Arabidopsis* plants. The first column is the Maker band and 1-12 are the transgenic strain bands, where 2,5 and 7 are the positive plant bands.
